# Systematic review of primary and booster COVID-19 sera neutralizing ability against SARS-CoV-2 omicron variant

**DOI:** 10.1038/s41541-022-00565-y

**Published:** 2022-11-15

**Authors:** Ioannis Sitaras, Henning Jacobsen, Melissa M. Higdon, William E. Dowling, Naor Bar-Zeev, Maria Deloria Knoll

**Affiliations:** 1 W. Harry Feinstone Department of Molecular Microbiology and Immunology, Johns Hopkins Bloomberg School of Public Health, Baltimore, MD USA; 2 Department of Viral Immunology, Helmholtz Centre for Infection Research, Braunschweig, Germany; 3 International Vaccine Access Center, Department of International Health, Johns Hopkins Bloomberg School of Public Health, Baltimore, MD USA; 4 Coalition for Epidemic Preparedness Innovations, Washington DC, USA

**Keywords:** Policy and public health in microbiology, Vaccines, SARS-CoV-2

## Abstract

Virus neutralization data using post-vaccination sera are an important tool in informing vaccine use policy decisions, however, they often pose interpretive challenges. We systematically reviewed the pre-print and published literature for neutralization studies against Omicron using sera collected after both primary and booster vaccination. We found a high proportion of post-primary vaccination sera were not responding against Omicron but boosting increased both neutralizing activity and percent of responding sera. We recommend reporting percent of responders alongside neutralization data to portray vaccine neutralization ability more accurately.

## Introduction

Since its initial characterization in November 2021, the SARS-CoV-2 Omicron (B.1.1.529) variant of concern (VOC) has largely displaced the Delta variant in many parts of the world^1–3^. The World Health Organization (WHO) and the Coalition for Epidemic Preparedness Innovations (CEPI) among others use information on the neutralizing activity of post-vaccination sera as an early sign of vaccine performance against circulating VOCs. Since neutralization data could inform policy-making decisions regarding vaccine strategies, we reviewed the global evidence for current vaccines’ ability to neutralize Omicron, and stratified the results according to primary vaccination series and booster (both of which included homologous and heterologous regimes).

## Results

### Included studies and number of sera

Neutralization results were extracted from 50 studies meeting our inclusion criteria, providing data for 119 vaccine-specific observations based on 3150 sera (72 observations on 1823 sera for primary vaccination series, and 47 observations on 1327 sera for booster vaccination, Table 1). Details of the included studies and raw data can be found in the Source Data file.

**Table 1:** Overview of studies and results.

Primary vaccination series									
			No. Obs	No. of Sera	Fold decrease (Omicron)			Median % responders (Omicron)	Median GMT (Parent)
Type of vaccination	Platform	Vaccine			Q1	Median	Q3		
Homologous	Inactivated	BBIBP-CorV	4	325	1.9	7.2	11.2	23	67.4
		CoronaVac	4	135	5.4	9.5	14.5	0	32.5
	mRNA	mRNA-1273	14	275	15.8	25.5	42.6	94	1039
		Comirnaty	33	777	18.9	23.1	34.7	38	347.9
	Vector	Vaxzevria	6	131	13.3	17.5	25	12	133
		Sputnik V	2	26	7.6	7.6	7.6	44.5	66.5
		Ad26.COV2.S	7	128	3.0	6.2	10.7	8.5	96
Heterologous	Vector + mRNA	Vaxzevria + mRNA-1273	1	11	19.5	19.5	19.5	82	671.4
		Vaxzevria + Comirnaty	1	15	24.5	24.5	24.5	80	661.6
Total			72	1823					
Booster									
Homologous	Inactivated	BBIBP-CorV (3 doses)	4	408	8.6	11.4	15.8	78	361.8
		CoronaVac (3 doses)	2	70	9.7	13.1	16.5	72.5	164.3
	mRNA	mRNA-1273 (3 doses)	7	74	4.2	5.5	15	100	3942
		Comirnaty (3 doses)	19	438	4	7.4	13.1	100	1749.5
	Protein subunit	Anhui ZL (3 doses)	3	44	3	9.4	10.6	56	329
	Vector	Sputnik V + Sputnik Light	1	6	7.1	7.1	7.1	100	359.2
		Ad26.COV2.S (2 doses)	1	20	2.8	2.8	2.8	100	127.6
Heterologous	Inactivated + Protein subunit	BBIBP-CorV + Anhui ZL	2	20	11.8	13.4	14.9	90	1527.5
	Inactivated + mRNA	CoronaVac + Comirnaty	2	85	6.1	6.7	7.3	100	320
	mRNA + mRNA	mRNA-1273 + Comirnaty	1	4	13.1	13.1	13.1	75	984.3
		Comirnaty + mRNA-1273	1	4	9.8	9.8	9.8	100	1378.6
	mRNA + Vector	Comirnaty + Ad26.COV2.S	2	61	2.9	3.6	4.2	100	2456.3
	Vector + mRNA	Ad26.COV2.S + Comirnaty	1	20	-	-	-	100	1044.9
	Vector + Protein subunit	Vaxzevria + MVC-COV1901	1	73	6.6	6.6	6.6	N/R	404.8
Total			47	1327					

Results for each vaccine regimen (primary series and booster), with median fold change against Omicron, IQR, and median GMT against the parent strain (which was chosen to be similar to the vaccine-seed strain). Results could not be extracted; N/R Not Reported. References ^4–53^ were used to collect the source data used in the analysis, which can be found in the Source Data file.

Most primary series and booster observations were of mRNA vaccines (Comirnaty primary: 33/72, booster: 19/47; mRNA-1273 primary: 14/72, booster: 7/47; Table 1). Vaccines with the largest number of sera evaluated were Comirnaty (primary: 777; booster: 438) and BBIBP-CorV (primary: 325; booster: 408). However, most BBIBP-CorV sera originated from one large study of 292 sera^4^. Most (87/119) observations evaluated sera collected 0.5–1 month post-vaccination.

### Results stratified by vaccine and regimen

**Fig. 1:**
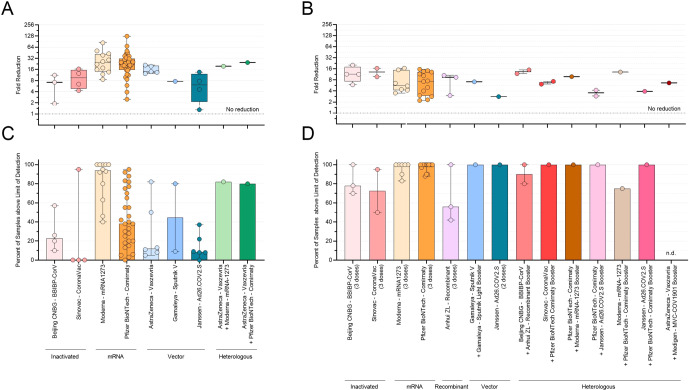
Primary vaccination series and booster neutralizing ability and percent responders against Omicron. Neutralizing ability of antibodies against Omicron variant by vaccine as measured by **A**: fold reduction post-primary series vaccination; **B**: fold reduction post-booster vaccination; **C**: percent of responders post-primary; **D**: percent of responders post-booster. Thick lines represent median, thin lines represent interquartile range (IQR).

Median fold decreases in Omicron neutralization post-primary vaccination series ranged widely across the vaccines studied, from 6.2-fold (Ad26.COV2.S, IQR 3.0–10.7, 7 observations, 128 sera) to 23.1-fold and 25.5-fold (Comirnaty: IQR 18.9–34.7, 33 observations, 777 sera; mRNA-1273: IQR 15.8–42.6, 14 observations, 275 sera Table 1, Fig. 1A). The percent of responders also ranged widely in primary vaccination series from 0% for CoronaVac to 94% for mRNA-1273 (Table 1 and Fig. 1C). Although the only two heterologous vaccine regimes (Vaxzevria+Comirnaty and Vaxzevria+mRNA-1273) showed 80 and 82% responders respectively, these results were based on only one observation from the same study^5^.

After a booster dose, median fold decreases in neutralization activity were lower compared to primary vaccination series, ranging from 2.8 (Ad26.COV2.S, 1 observation, 20 sera) and 13.4 (BBIBP-CorV+Anhui ZL heterologous boost, IQR 11.8–14.9, 2 observations, 20 sera, Table 1, Fig. 1B). The percent of responders increased post-booster for all vaccines, with 12 of the 14 booster regimens examined having >70% responders (8 of which showed 100% response). From the two remaining regimens, Anhui ZL had a 56% response, but the percent response could not be extracted for the one study describing the Vaxzevria+MVC COV1901 regimen (Table 1 and Fig. 1D). The percent of responders correlated with the GMT against the parent strain, which was significantly higher in booster regimes compared to primary series (Table 1).

The median fold decreases against Omicron were generally comparable within the same vaccine class for mRNA (23.1 and 25.5 post-primary; 5.5 and 7.4 post-booster) and for inactivated vaccine classes (7.2 and 9.5 post-primary; 11.4 and 13.1 post-booster) but varied within vector vaccine class (range: 6.2 to 17.5 post-primary, 2.8 and 7.1 post-booster, Table 1).

## Discussion

In this review of COVID-19 vaccines’ abilities to neutralize the Omicron variant, we show that booster doses increased neutralizing activity compared to primary series vaccination, as evidenced by lesser fold decreases and greater percent of responding sera. The increased activity following the booster dose cannot be explained by prior immune waning, since the vast majority (73%) of included studies evaluated sera collected at peak immunity. We also demonstrate that relying only on fold decrease when the percent of responding sera is low can mask the true neutralizing activity of a vaccine, and, consequently, may lead to inaccurate inference of a vaccine’s ability to protect against infection and disease from variants, especially one as antigenically different as Omicron.

The percent of responding sera is an overlooked but highly important parameter in assessing post-vaccination neutralizing activity against variants. The accuracy of fold decrease calculations can be misleading when the majority of sera show no measurable neutralizing activity against Omicron. This is because when serum measurements are below the limit of detection, the general practice is to assign them an arbitrary titer, which is usually either one log lower than the lowest dilution, or half the level of detection. This arbitrary value can have a large impact on the magnitude of the apparent fold change, since it can over- or underestimate neutralizing activity against a variant. It is thus important to also report the percent of responding sera to provide a more complete and accurate picture of a vaccine’s ability to elicit neutralizing immunity against variants.

The lower limit of detection varies greatly between assays that use live viruses *versus* pseudoviruses. It is important to note, however, that although very low readings can be obtained when using pseudoviruses, the uncertainty connected to these readings increases proportionally the lower the reading is. This holds true for every reading obtained that is already below the lowest (starting) dilution used in the assay. Our suggestion to include the percent of responding sera in the results, helps to eliminate part of this uncertainty.

Induction of high neutralizing titers against the homologous vaccine-seed strain is not only part of regulatory requirements, but also an important early measure of a vaccine’s success, since higher titers generally correspond to higher levels of protection, including against future circulating variants as antigenically different as Omicron. Our analysis shows that GMTs against the parent strain are lower after the primary vaccination series than after the booster dose and corresponded with lower percent of responding sera against Omicron after primary vaccination compared to post-booster dose.

Although some vaccines and vaccine combinations appear to perform better than others, the wide heterogeneity in responses between studies observed for some homologous vaccines mean these data should be considered with caution. This is even more true for all heterologous vaccine combinations evaluated here, where data on their neutralizing ability were available from only 1–2 studies each, and therefore these results may not represent their true abilities. Nevertheless, such data are especially important in the current setting, where reliable clinical effectiveness data may not be available for all vaccines in use, and where administration of heterologous doses can be common because of supply or logistics issues. If meaningful appraisals of vaccine performance are to be made in order to inform timely policy-making decisions, there is an urgent need for more data on the vaccines currently in use. Using the WHO international standard (IS) and reporting results in international units (IU) could in theory allow for better comparison between assays. However, considering that the WHO IS has largely been depleted, and is found to have no neutralizing activity against Omicron variants, reporting the results in IU is not feasible.

A possible limitation of our analysis may come from the inclusion of data from pre-printed articles, which should always be used with caution. Nevertheless, the use of these articles does not affect the main findings and recommendations of this manuscript, namely that neutralizing ability and % of responders against Omicron increase post-boost compared to primary vaccination series, and that there is a need for more data for certain vaccines and vaccine combinations. Both findings hold true regardless of the source of data (published or otherwise). Finally, using pre-prints in order to inform policy-making decisions regarding vaccine strategies has become acceptable during the pandemic, due to urgency of the situation and the need to respond and adjust policies as quickly and effectively as possible. Restricting the analysis to fully published literature would have resulted in the exclusion of relevant and useful data that could help inform our understanding of the impact of Omicron on vaccine performance.

## Methods

### Literature search, review, and exclusion criteria

This work consists of analysis of data available in the public domain. No ethics approval is necessary.

**Fig. 2:**
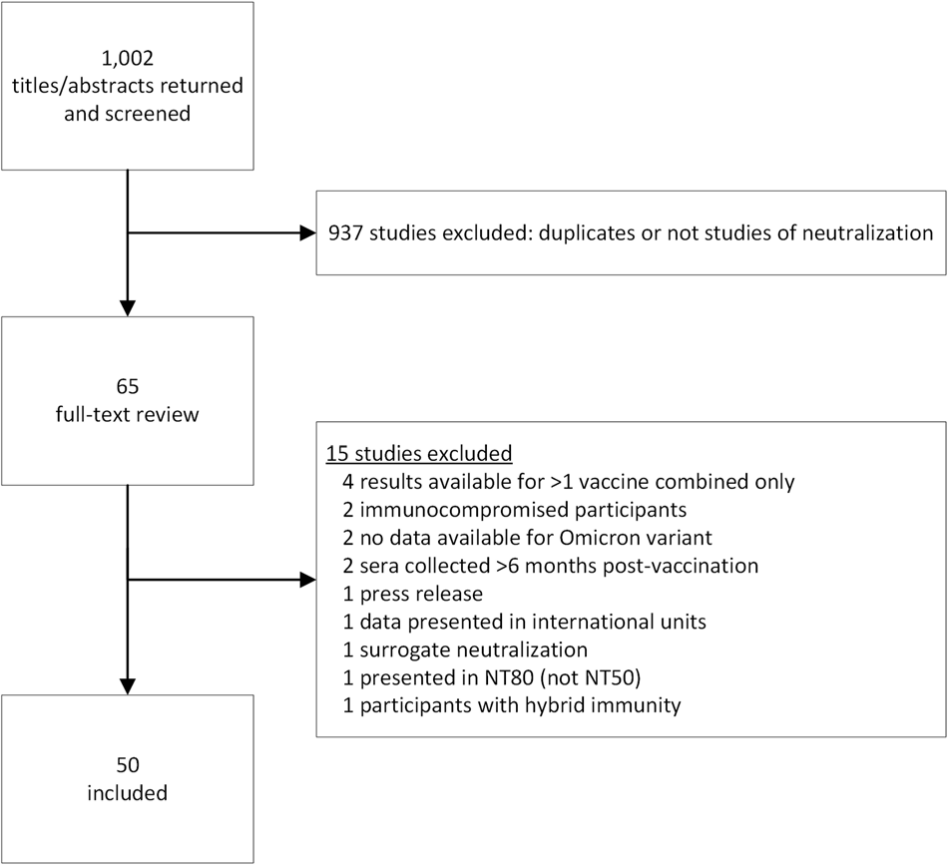
PRISMA flow diagram. Overview of the systematic literature search and study selection process.

Studies containing neutralization data against the Omicron variant after primary series or booster vaccination were identified by searching PubMed and pre-print servers (bioRxiv and medRxiv) from November 1, 2021 until January 31 2022 using the following terms: “SARS-CoV-2” AND “Omicron” AND (“neutralization” or “neutralization”). Title and abstract were screened and those possibly containing relevant data underwent full text review. Relevant articles underwent data abstraction in real-time. Exclusion criteria included: sera collected after partial primary series vaccination; sera collected <7 days or ≥6 months post-vaccination; data collected without using a strain genetically/antigenically similar to the vaccine-seed strain as a comparison (i.e., Wuhan, WA-1, or the D614G strain); data from subjects infected with SARS-CoV-2 either before being vaccinated or after; data from cohorts that included immunocompromised individuals, or individuals with concurrent health conditions that are expected to affect vaccination-induced immunity (for example cancer patients). In addition, data from assays using pseudoviruses or virus-like particles were excluded if these viruses did not contain the full complement of mutations characteristic of the Omicron VOC spike protein. If any studies did not contain clear information on any of the exclusion criteria, they were not included in the analysis. Studies were screened by three reviewers working independently and collaborating when in doubt. A PRISMA diagram describing the literature search and study selection process is shown in Fig. 2. A review protocol was not prepared.

### Data extraction and analysis

The fold change in neutralizing activity against Omicron (relative to the parent strain), the geometric mean titer (GMT) of the parent strain, and the percent of responding sera (defined as sera with a neutralizing activity against Omicron above each study’s lower limit of detection) were abstracted for each vaccine regimen. All references used to collect the source data can be found in the reference section of this manuscript^4–53^, as well as in the Source Data file. Raw data can be found in the Source Data file. All neutralization data used were reported as NT_50_ titers. Median and interquartile ranges (IQR) of the fold decrease in neutralization activity, median percent responders, and median parent strain GMTs across all studies were calculated where possible (using the GMTs reported in each source reference) for each unique vaccine combination and plotted using GraphPad Prism 9 (version 9.3.1). Meta-analyses to estimate average effect size were not performed due to heterogeneity in the methodology of included studies.

### Reporting summary

Further information on research design is available in the Nature Research Reporting Summary linked to this article.

## Supplementary information

Data Set 1

REPORTING SUMMARY

## Data Availability

All data generated or analyzed during this study and underlying Fig. 1 are included in this published article and its Source Data file.
